# Comment on Retuerto et al. Analysis of Gut Bacterial and Fungal Microbiota in Children with Autism Spectrum Disorder and Their Non-Autistic Siblings. *Nutrients* 2024, *16*, 3004

**DOI:** 10.3390/nu17233681

**Published:** 2025-11-25

**Authors:** Tatsuki Itagaki, Ken-ichiro Sakata, Jun Sato, Akira Hasebe

**Affiliations:** 1 Oral Diagnosis and Medicine, Faculty of Dental Medicine, Graduate School of Dental Medicine, Hokkaido University, Kita-13 Nishi-7, Kita-ku, Sapporo 060-8586, Japan; titagaki@den.hokudai.ac.jp (T.I.); sakata-0303@den.hokudai.ac.jp (K.-i.S.); jun-s@den.hokudai.ac.jp (J.S.); 2 Microbiology, Faculty of Dental Medicine, Graduate School of Dental Medicine, Hokkaido University, Kita-13 Nishi-7, Kita-ku, Sapporo 060-8586, Japan

We would like to comment on the recently published article by Retuerto et al. (2024) entitled “Analysis of Gut Bacterial and Fungal Microbiota in Children with Autism Spectrum Disorder and Their Non-Autistic Siblings” [1]. We are also conducting research on microbiota in children with autism spectrum disorder (ASD). We have some concerns about their findings and paper. We would like them to consider the results from a different viewpoint.

Principal component analysis (PCA) and principal coordinate analysis (PCoA) cannot be used usually at the same time. In other words, only PCoA or PCA can be used to reduce the dimension of a single data set, and the two methods cannot be used on the same data. In our conclusion, PCA using sample variance is optimal for bacterial flora analysis of relative abundance [2]. Moreover, non-parametric Spearman correlation and a Wilcoxon rank-sum test were used in the research. The operational taxonomic units are standardized for each sample, so non-parametric tests cannot be applied since it is necessary to use overall ranks.

As the abstract states, “Differences between ASD and non-ASD subjects include a significant decrease at the phylum level in Cyanobacteria (0.015% vs. 0.074%, *p* < 0.0003), and a significant decrease at the genus level in Bacteroides (28.3% vs. 36.8%, *p* < 0.03) [1]”. We should understand the difference between relative abundance, or percentage, and absolute abundance. A high percentage does not necessarily mean a high absolute abundance. Although the paper states that there is a “significant decrease at the genus level in Bacteroides (28.3% vs. 36.8%)”, the total amount of bacteria generally varies among individuals, so the absolute amount may be 28.3% higher. For example, when the total amount of bacteria is 1000 and 700, the absolute amount is 283 (1000 × 28.3%) and 258 (700 × 36.8%), respectively, and the relative abundance and absolute abundance are reversed.

Many believe these are incorrect statistical methods but correct results. Compositional data only allow comparison of the same components. Results that result from a breakdown in logic are not scientific. We seek the truth. Therefore, we recommend using centered logratio transformation or additive logratio transformation for microbial compositional data [2]. If there is a component whose absolute amount remains constant, the increase or decrease in absolute amount can be determined using additive logratio transformation from only the relative abundance [2].

In summary, Retuerto et al. should reanalyze using other methods and viewpoints. We look forward to seeing the results of Retuerto et al.’s reanalysis to see whether they are consistent with our results (unpublished).
